# Supplementary material for: Predicting genetic biodiversity in salamanders using geographic, climatic, and life history traits
Source: PLoS One. 2024 Oct 18;19(10):e0310932. doi: 10.1371/journal.pone.0310932 (PMC11488749; doi:10.1371/journal.pone.0310932)
Supplement: S1 Table — (DOCX) [file pone.0310932.s002.docx]

| **Gene** | **Num sequences** | **Num species** |
| --- | --- | --- |
| 12S | 4 | 1 |
| 16S | 105 | 15 |
| 18S | 12 | 2 |
| BDNF | 22 | 1 |
| CALR | 347 | 2 |
| COI | 800 | 81 |
| CYTB | 1091 | 29 |
| NCX1 | 24 | 1 |
| ND1 | 8 | 2 |
| ND2 | 485 | 5 |
| ND4 | 817 | 9 |
| POMC | 24 | 1 |
| RAG1 | 16 | 2 |
| TRCA | 13 | 1 |

**Table S1.** Comparison of salamander data available for each loci on the phylogatR database. Table displays the number of sequences present in each gene alignment and the number of described species covered by the specific gene.
