# Supplementary material for: Predicting genetic biodiversity in salamanders using geographic, climatic, and life history traits
Source: PLoS One. 2024 Oct 18;19(10):e0310932. doi: 10.1371/journal.pone.0310932 (PMC11488749; doi:10.1371/journal.pone.0310932)
Supplement: S2 Table — (DOCX) [file pone.0310932.s003.docx]

| Clade | Model | Accuracy (95% CI) Length |
| --- | --- | --- |
| Caudata | MR original | 0.3694 |
|  | MR \|Correlation\| > 0.75 | 0.3694 |
|  | MR \| Correlation \| > 0.85 | 0. 3694 |
|  | MR \| Correlation \| > 0.90 | 0.3264 |
|  | AA Original | 0.388 |
|  | AA \|Correlation\| > 0.75 | 0.3178 |
|  | AA \| Correlation \| > 0.85 | 0.388 |
|  | AA \| Correlation \| > 0.90 | 0.388 |
| Mammalia | ABGD COI | 0.1083 |
|  | ABGD cytb | 0.0908 |
|  | GMYC COI | 0.1183 |
|  | GMYC cytb | 0.0982 |
|  | Consensus | 0.1012 |

**Table S2.** Comparison of model accuracy confidence intervals between salamander and mammal predictive models. In the clade Caudata, MR indicates majority rules models and AA indicates all agree models. Mammal data is summarized from Parsons et al. 2022.
